# The Impact of Transformational Leadership on Affective Organizational Commitment and Job Performance: The Mediating Role of Employee Engagement

**DOI:** 10.3389/fpsyg.2022.831060

**Published:** 2022-04-06

**Authors:** Wang Jiatong, Zheng Wang, Mehboob Alam, Majid Murad, Fozia Gul, Shabeeb Ahmad Gill

**Affiliations:** ^1^College of Teacher Education, Zhejiang Normal University, Jinhua, China; ^2^China University of Political Science and Law, Beijing, China; ^3^Treasurer Office, Lahore College for Women University, Lahore, Pakistan; ^4^School of Management, Jiangsu University, Zhenjiang, China; ^5^The Institute of Management Sciences, PAK AIMS, Lahore, Pakistan; ^6^Lyallpur Business School, Government College University, Faisalabad, Pakistan

**Keywords:** transformational leadership, employee engagement, affective organizational commitment, job performance, hospitality sector, China

## Abstract

This study investigated the impact of transformational leadership on affective organizational commitment and job performance with the mediating role of employee engagement. This study gathered data from 845 hotel employees in China and the structural equation modeling technique was used to verify the results. The findings indicated that transformational leadership has a positive effect on affective organizational commitment and job performance. Meanwhile, results showed that employee engagement partially mediates in the relationship between transformational leadership, affective organizational commitment, and job performance. This study contributes to the research on transformational leadership in the Chinese hospitality sector and analyzes its effects on work performance metrics. Furthermore, theoretical and practical implications were also discussed in this article.

## Introduction

Leadership plays an important role in growing organizations and individual job performance (Qi et al., 2019; Afsar et al., 2020). In particular, a leader must provide his/her followers with what they need to be effective and move toward a common vision (Ahmad, 2018; Zhao and Zhou, 2019). Transformational leadership represents a leadership style that covert subordinates to see beyond self-interest by changing their confidence and interest to perform beyond expectations (Jena et al., 2018; Khan et al., 2021c). Transformational leadership is a crucial element for organizations that are willing to anticipate fundamental transitions to have an adequate environment for positive or adaptive changes through the effective process (Khan et al., 2020a; Hai and Park, 2021). Leaders’ action as transformational acting as learning support is nearly linked to the culture of affective organizational commitment (Cho et al., 2019; Mwesigwa et al., 2020).

Looking into previous literature the authors have examined the studies on transformational leadership and organizational performance (Katou, 2015), employee innovative behavior (Pieterse et al., 2010; Choi et al., 2016), and counterproductive work behavior (Kessler et al., 2013; Huang et al., 2021) in the context of banking, education, and health sectors. The research on the hospitality industry is empirically less explored. Hospitality employees are regarded as the most unique and valuable asset and the most costly investment of the hotel entities (Hwang et al., 2021). In the developing countries, such as Korea, China, Vietnam, and Thailand, hospitality jobs do not seem attractive to high-quality personnel (Wang and Abukhalifeh, 2020) due to several limitations, such as low wage (Wen et al., 2018), few career promotion opportunities, and high physical and/or emotional stress from work (e.g., long periods of standing or sitting, dealing with many customer complaints). In addition, hospitality jobs do not merit a high social status, unlike high-tech jobs, in society at large (Tuan, 2021).

Prior studies demonstrated the role of authentic (Niu et al., 2018), servant (Karatepe et al., 2020), and transformational leadership on job satisfaction and innovative work behavior (Li et al., 2020a; Khan et al., 2021a). According to Schuckert et al. (2018), transformational leaders are capable of allowing individuals to work collectively and transcend their self-interests through many transformational leadership dimensions such as charisma, intellectual stimulation, individualized consideration, and inspirational motivation. Transformational leadership holds an important and well-built impact on affective organizational commitment (Yi et al., 2019).

Existing studies remarked the activist part of transformational leadership on job attitude and proactive behavior (Steinmann et al., 2018) and a few studies try to identify the relationship between transformational leadership, employee engagement, and affective organizational commitment. Transformational leadership provides a positive result to employee job performance (Khan and Khan, 2019), as transactional leadership looks too weak commitment of employee and their satisfaction (Cho et al., 2019), discovering transformational leadership predicting to examine the performance and work-related helpful behaviors are critical for employees of the hospitality industry (Liang et al., 2017; Moin et al., 2020). A few articles were found in journals of hospitality that examined the relationship between transformational leadership, employee engagement, affective organizational commitment, and job performance (Gui et al., 2020).

Besides, research specifies the characteristics of transformational leaders such as idealize intelligent stimulus and individualized reflection are significantly associated with intellectual, expressive, and social engagement of employees (Prikshat et al., 2020). The existing study indicated that transformational leadership and employee engagement had a significant effect on job performance with the mediating role of employee engagement (Khan et al., 2020b). Yet, few empirical studies have examined to explore in which way transformational leadership could enhance the intensity of employee engagement that gives affective organizational commitment (Luo et al., 2019; Katou et al., 2020). Therefore, there is a research need to predict the influence of transformational leadership on the employee to improve their job performance in the hospitality sector of China.

This study contributes to the literature in the following perspectives; at first existing research indicated employee engagement holds a crucial role to control the behavior of leaders on subordinate attitude and performance (Steinmann et al., 2018; Khan et al., 2021b). Second, employee engagement as a mediator helps employees to improve their performance to achieve a competitive advantage. Thirdly, prior research indicated that there is a lack of research that explores transformational leadership on job performance and affective organizational commitment in the literature of Asian context (Ribeiro et al., 2018; Sungu et al., 2019). Accordingly, this study aims to investigate the impacts of transformational leadership on job performance and affective organizational commitment, and also the mediating effect of employee engagement on their relationships.

## Theory and Hypotheses Development

### Theoretical Support

Numerous researchers argued that the role of the job demands—resources model and social exchange theory (SET) in transformational leadership is to make the casual relationship between subordinate and leader for the building of shared harmony (Cho et al., 2019; Katou et al., 2021). Employee engagement and intensity of their affective organizational commitment are linked to support transformational leadership and their ideas to enhance job performance (Sungu et al., 2019). Existing research revealed that the constructive link of transformational leadership to the attitude and performance of an employee is positively associated with each other (Cho et al., 2019). Transformational leadership is a vital channel for the encouragement of affective organizational commitment of employees and job performance with the mediation role of employee engagement.

### Hypotheses Development

#### Transformational Leadership and Affective Organizational Commitment

Transformational leadership has been identified as an important contributing factor in the development of affective organizational commitment (Sahu et al., 2018). The relationship between the leader and follower has been developed when transformational leaders use individual consideration to meet follower’s needs, transcend economic transactions, and contribute to long organizational tenure and strong commitment (Nazir and Islam, 2017). Previous researchers indicated that transformational leadership is the best predecessor of affective organizational commitment (Islam et al., 2018). Affective organizational commitment describes “the state of emotional connection so that committed employee recognizes with, concerned and take pleasure of being of the organization”(Benevene et al., 2018; Rodrigo et al., 2019). Affective organizational commitment is more linked than normative or continuance commitment to the organization and employee’s relevant outcomes because it is related to an individual’s intrinsic motivation, whereas normative and continuance commitment is associated with the feeling of obligation or pressure (Kim and Beehr, 2018).

Wong and Wong (2017) demonstrated the relationship between transformational leadership and affective organizational commitment. From the Chinese perspective, an intellectual leader holds more power on affective commitment as compared with the continuance commitment. Chinese affective commitment is being considered as the sole gauge for organizational commitment (Nazir et al., 2018). The existing study argued that there is a positive correlation between transformational leadership and affective organizational commitment (Buil et al., 2019). Hence, this study hypothesized;

**H1:** Transformational leadership has a positive effect on affective organizational commitment.

#### Transformational Leadership and Job Performance

The relationship between transformational leadership and job performance was examined by prior researchers (Darvishmotevali and Ali, 2020). To enhance employee performance, transformational leadership support employees for persistence and sympathy in work duties (Schwarz, 2017). Transformational leaders help to satisfy employees at work psychologically and keep them happy, resultantly enhancing employees’ job performance (Lai et al., 2020). Transformational leadership defines as proactive behavior, raising awareness of the common interests of followers, and helping followers achieve goals at the highest level. Transformational leadership is a leader who can inspire, motivate, and give a great influence to his followers to do more work than expected and put aside personal interests for the benefit of the organization (Eliyana et al., 2019).

An existing study discovered the constructive relationship among transformational leadership, employee innovative behavior, and attitude regarding perceived results (Buil et al., 2019). Furthermore, as compared with the other leadership styles such as ethical leadership, authentic leadership, servant leadership, transformational leadership had a significant influence on employee’s performance (Hameed et al., 2020), because transformational leadership holds manifold characteristics including moral values (Rodriguez et al., 2017; Buil et al., 2019). Transformational leadership has proved to be an important tool for the employees.

From the Chinese organizational perspective, the transformational leadership constructs reliability face issues regarding the traditional way of leadership styles because of the quickly changing work environment (Le and Lei, 2017). Therefore, in a Chinese context, transformational leadership is considered a vital medium to encourage the attitude of an employee and their performance. Transformational leadership theory describes that the vision needs to be communicated by the leader, who needs to motivate and inspire the employees. Leaders are effective when they involve their employees in achieving the company’s vision by trusting and involving them in goal setting. The focus of a leader should be to support the employees and to encourage critical thinking (Dai et al., 2013). Therefore, the following hypothesis is predicted;

**H2:** Transformational leadership has a positive effect on employee job performance.

#### Transformational Leadership and Employee Engagement

Prior research has discussed the behavior of transformational leadership and its impact on the degree of employees engagement (Li et al., 2019; Mi et al., 2019; Yang et al., 2020). The previous study revealed that transformational leadership significantly influenced job engagement (Bui et al., 2017). A transformational leader can encourage employee engagement, defined as an individual employee’s cognitive, emotional, and behavioral state directed toward desired organizational outcomes, by fostering his/her subordinates’ positive behaviors and attitudes toward work, and also supporting their self-efficacy to the challenging vision and goal (Lai et al., 2020).

Transformational leadership behaviors are stimulating employee engagement and provide a clear vision that can help employees to internalize the organization’s goals and understand how valuable their contributions are toward achieving this vision (Schwarz, 2017). Subsequently, the employees may become engaged because they are certain about the connections between their efforts and the future of their organization. Intellectual stimulation involves leaders challenging employees to critically examine situations and find creative solutions to organizational problems (Buil et al., 2019). Such leader behavior may not only influence employees’ perceptions that the job is more challenging but also their perceptions of autonomy in the work climate because employees are given the flexibility to solve problems using novel methods (Hameed et al., 2019). Taken together, increased perceptions of challenge and autonomy can activate employees intrinsically, and thus, may increase employee engagement (Koroglu and Ozmen, 2021). Therefore, we hypothesized;

**H3:** Transformational leadership has a positive effect on employee engagement.

#### Employee Engagement and Affective Organizational Commitment

The studies conducted in the Western context demonstrated a positive relationship between employee engagement and affective organizational commitment (Allen and Meyer, 1990). Besieux et al. (2015) argued that with the enhancement of employee engagement and organizational commitment gets heightened. Furthermore, the job demand-resource model explains that engagement at work is much more effective than job demand in predicting organizational commitment since the latter predisposes an employee toward more professional and emotional exhaustion and ultimately leads to burnout (Jena et al., 2018). The previous investigation also indicates that a work environment that provides psychological safety and meaning ensures the commitment of the employees toward their organizations (Koroglu and Ozmen, 2021).

An existing study found that helpful relation of employee engagement is positively associated with affective commitment (Johnson et al., 2018; Srivastava and Singh, 2020). Meanwhile, research directed in the hospitality sector confirmed that corporate social responsibility and employee engagement are the positive antecedent of affective organizational commitment (Nazir and Islam, 2020). Most of the studies on employee engagement and organizational commitments are predominantly based on the Western organizational setup and have used Western samples (Buil et al., 2019). In the Indian context, Srivastava and Singh (2020), have tested the factors of commitment on the ground of performance among employees to understand the level of engagement among their executives. Besieux et al. (2015) described engagement as a form of commitment that is determined by the number of mutual efforts placed by the employees and the organizational development. Thus, the following hypothesis is predicted;

**H4:** Employee engagement has a positive effect on affective organizational commitment.

#### Employee Engagement and Job Performance

Employee engagement has been defined in different ways depending upon the context, in which it has been discussed. The definition of engagement revolves around specific attributes of the employee attitude (involvement, loyalty, and commitment) and employee behavior (such as taking initiatives and productivity levels) (Young et al., 2018). Many researchers identified employee engagement as a “psychological condition in work with three features including emotional, cognitive, and behavior vigor”(Albrecht et al., 2018; Kwon and Kim, 2020). Prior scholars argued that employee engagement is a positive indicator that impacts job performance (Gupta, 2015; Nazir and Islam, 2017). Engaged employees are very much attached to their work duties and subordinate that ultimately bring employee performance (Sahu et al., 2018).

Employees are capable enough to perform in the extra role when they are engaged. Albrecht et al. (2018) provided a comprehensive definition of employee engagement “as a positive, fulfilling work-related state of mind and is characterized by vigor, dedication, and absorption.” Vigor is described as a high level of energy, while dedication is described as a mental resilience that involves being strongly involved in one’s work and to experience a sense of significance, enthusiasm, and absorption is depicted as bringing a concentrated and engrossed self in employees’ performance (Le and Lei, 2017). Employee engagement at work is a better predictor of their cognitive, emotional, and behavior output, as it depicts their efforts toward the organization’s objectives by making them engage in a better way (Reilly, 2018; Alagarsamy et al., 2020). Thus, this study offers a subsequent hypothesis;

**H5:** Employee engagement has a positive impact on job performance.

#### Mediating Role of Employee Engagement

Based on the job demands-resource (JD-R model) an employee’s engagement is enhanced by job resources and it can produce positive work outcomes (Breevaart and Bakker, 2018; Kwon and Kim, 2020). Based on this JD-R model employee engagement has a mediating role in the relationship between job resources (Koroglu and Ozmen, 2021). The behavior of leaders in the shape of transformational leadership indicates job resources toward employee engagement (Katou et al., 2021). Radic et al. (2020) explained that job demand and resources impact the engagement of employees which in turn manipulates commitment toward the organization. Similarly, prior studies found that process of motivation like an instrument affects transformational leadership and performance through employee engagement (Besieux et al., 2015; Jena et al., 2018). Sahu et al. (2018) examined employee engagement as a mediating construct in the connection between transformational leadership and turnover intention. Buil et al. (2019) established the view of employee engagement is playing a mediation role in the association between transformational leadership and job performance. Hence, we predicted the following hypotheses:

**H6:** Employee engagement positively mediates the relationship between transformational leadership and affective organizational commitment.
**H7:** Employee engagement positively mediates the relationship between transformational leadership and job performance.

## Materials and Methods

**FIGURE 1:**
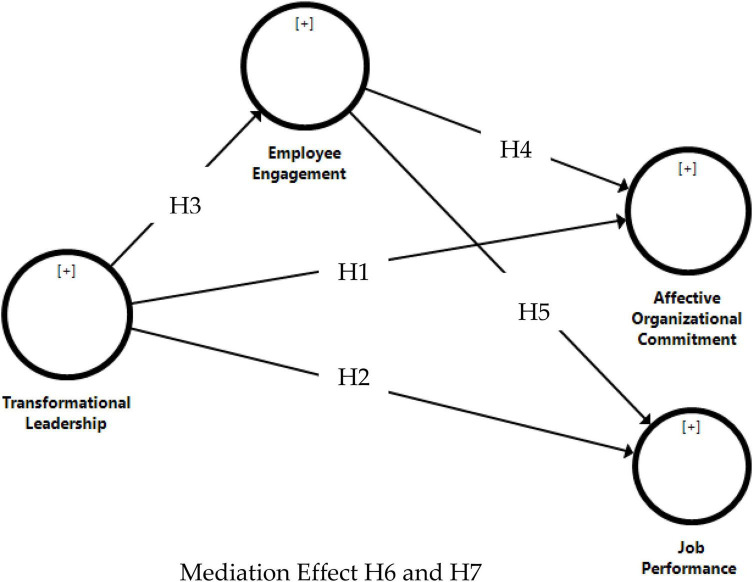
Conceptual model.

The conceptual model depicting the relationships and hypothesis is given in Figure 1.

### Sample and Data Collections

The nature of this study was cross-sectional and data were gathered from medium to upscale level (2–3 stars) hotels of Jiangsu and Zhejiang provinces of China. The non-probability convenience sampling technique was used because it was convenient for researchers to get data from respondents. Before going to collect data, an official email and consent letter was sent to the human resource department of the hotels. After the approval from the HR department, we visited hotels and gathered data from employees who expressed interest in participating to complete the questionnaire onsite. The data collection procedure was hectic and there was an issue of language communication, therefore, we took help from ten Chinese graduate students to facilitate the researchers in the data collection process. To secure the anonymity and confidentiality of the data, we ensured the participants that their answers were treated solely and not used for any other purpose. Moreover, we distributed 1,000 paper–pencil questionnaires among hotel staff and 900 completed surveys obtained. After the initial screening with the help of statistical software 55 questionnaires were discarded because of the incomplete forms of responses. Thus, 845 valid responses were incorporated for further analysis. The participants were (58.6%) male and (41.4%) female. Also, the highest number of the respondents (57.0%) was aged between 18 and 30 years old, with (77.0%) of them married. The majority of the respondents (59.3%) had a hospitality and hotel management diploma degree and (49.8%) of the participants were working in the hotel industry since 3 years.

### Instrument Development

The questionnaire was divided into two parts. The first part comprised of transformational leadership, employee engagement, affective organizational commitment, and job performance. The transformational leadership scale was based on (Dai et al., 2013) scale. It comprises of 8 items. An example item was “my supervisor encourages me to think about problems from a new perspective.” The Cronbach’s alpha for transformational leadership was (0.960). Moreover, employee engagement was measured using 7 item scale used by Wang et al. (2020). A sample item “I am proud of the work that I do.” The Cronbach’s alpha for employee engagement was (0.967). Affective organizational commitment was assessed using 6 items scale and adopted from the study of Meyer and Allen (1997). A sample item “I feel the problems of this organization as my problems.” The Cronbach’s alpha for affective organizational commitment was (0.911). Job performance was measured using 5 items scale and adopted from the study of Ozer (2011). A sample item “performs tasks that are expected of him/her.” The Cronbach’s alpha for job performance was (0.917). All the measurement constructs were rated using seven-point Likert scales ranging (from 1 = strongly disagree to 7 = strongly agree) and these measures are already tested and validated by others researchers (Li et al., 2020b; Shahzad et al., 2020). Furthermore, the second part contained the demographical information of the participants such as gender, age, education, marital status, and experience.

## Results

### Data Analysis Technique

The data were analyzed using the smart-partial least squares (PLS) software version 3.0. This software is currently considered as one of the suitable software to apply PLS structural equation modeling (PLS-SEM) (Sarstedt et al., 2014). PLS-SEM was recommended in most business management studies (Ali et al., 2018; Hair et al., 2019). This method is preferred for theory testing and confirmation and is appropriate for checking the existence of complex relationships. PLS-SEM allows the construction of a research paradigm based on a theory that involves transforming theories and concepts into unmeasured variables (latent) and practical concepts into metrics, all of which are connected by a theory or hypothesis (Ringle et al., 2014). Hair et al. (2012) recommended that the PLS-SEM model should be assessed in three phases: identifying the global model assessment, checking the measurement model’s validity, and analyzing the relevance of the routes inside the structural equation model.

### Measurement Model

To test the reliability of constructs the Cronbach’s alpha, composite reliability (CR) and average variance extracted (AVE) values were assessed. According to Henseler and Fassott (2010), the criterion for ensuring the CR is that all the values must be higher than 0.80. All the values of CR are given in Table 1 and lie between the ranges of 0.931–0.973, which confirms the CR of all of the constructs. Moreover, the Cronbach’s alpha for all he constructs was also above the threshold value of 0.70 suggested by Hair et al. (2011). Furthermore, the AVE criterion allows its value to be greater than 0.50 (Bagozzi and Yi, 1988). Therefore, Table 1 shows that the AVE values of the constructs were ranged from 0.692 to 0.838 and met the criteria. Prior researchers argue that if the values of AVE are above than an acceptable level of 0.50, it indicates adequate convergent validity.

**TABLE 1:** Measurement model.

Variable and constructs	Loadings	α	rho_A	CR	AVE	VIF
**Transformational leadership**		0.960	0.960	0.967	0.784	
TL1	0.944					2.886
TL2	0.815					2.450
TL3	0.920					2.675
TL4	0.859					3.426
TL5	0.836					4.274
TL6	0.927					3.160
TL7	0.941					2.780
TL8	0.832					3.550
**Employee engagement**		0.967	0.968	0.973	0.838	
EE1	0.817					4.412
EE2	0.933					3.093
EE3	0.945					2.566
EE4	0.944					4.579
EE5	0.956					3.468
EE6	0.956					2.102
EE7	0.846					2.742
**Affective organizational commitment**		0.911	0.914	0.931	0.692	
AC1	0.848					2.676
AC2	0.849					2.573
AC3	0.835					2.270
AC4	0.798					2.175
AC5	0.818					2.244
AC6	0.843					2.756
**Job performance**		0.917	0.920	0.938	0.752	
JP1	0.850					2.473
JP2	0.849					2.598
JP3	0.864					2.854
JP4	0.886					3.140
JP5	0.884					3.039

*α, Cronbach’s alpha; CR, Composite Reliability; AVE, Average Variance Extracted; VIF, Variance inflation factor. TL, Transformational leadership; EE, Employee engagement; AC, Affective organizational commitment; JP, Job performance.*

In addition, discriminant validity was calculated using the Fornell–Larcker criterion as findings are shown in Table 2. The findings show that constructs’ correlations with each other and below the square roots of their AVE (Fornell and Larcker, 1981). Besides, the discriminant validity was also assessed using heterotrait-monotrait (HTMT) criteria (Hair et al., 2011). Table 3 results indicate that HTMT values were satisfactory and below the threshold of 0.85 as suggested by Henseler and Fassott (2010). We also calculated the variance inflation factors (VIFs) for all constructs in our model to test for multicollinearity. All the VIF values were below 3.525, lower than the threshold of 5, indicating no concerns regarding multicollinearity issues in the data (Sarstedt et al., 2014). Finally, Harman’s single factor test was used to check for common method bias in the data. According to Harman’s technique, common method bias exists when one factor emerges from factor analysis and explains more than 50% of the variance (Podsakoff et al., 2003). We used the rotated solution to transfer all of the items into a one-factor analysis, yielding four factors; the first factor’s eigenvalue explains 30.61% of the variance (less than 50%). As a result, this study does not have an issue of common method bias.

**TABLE 2:** Table Fornell–Larcker criterion.

	AC	EE	JP	TL
AC	0.832			
EE	0.391	0.915		
JP	0.491	0.389	0.867	
TL	0.408	0.422	0.465	0.886

*TL, Transformational leadership; EE, Employee engagement; AC, Affective organizational commitment; JP, Job performance. Items with diagonals are the square root of the AVE. Items under diagonals are the correlations.*

**TABLE 3:** Heterotrait-monotrait (HTMT) criterion.

	AC	EE	JP	TL
AC				
EE	0.413			
JP	0.534	0.408		
TL	0.543	0.436	0.494	

*TL, Transformational leadership; EE, Employee engagement; AC, Affective organizational commitment; JP, Job performance.*

### Structural Model

**FIGURE 2:**
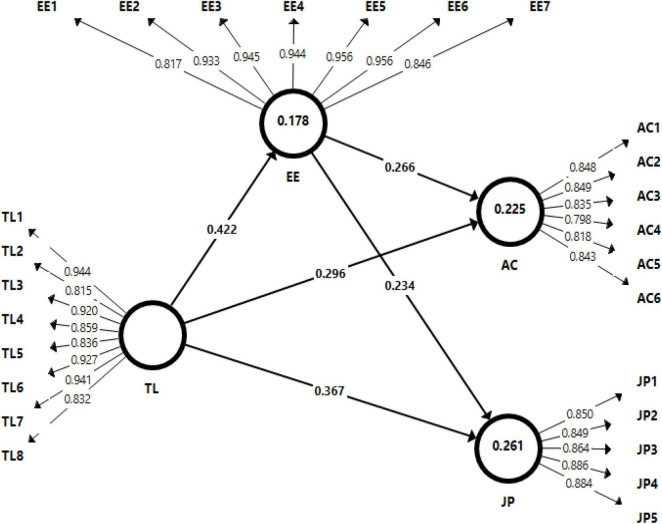
Measurement model.

The structural model was evaluated through the 5,000 bootstrap method with the help of Smart-PLS software. The fitness of the structural model was assessed by the standardized root mean squares residual (SRMR) value. According to Henseler and Fassott (2010), a good structural model should have below the 0.08 SRMR value. Therefore, the findings from the structural model show a (0.049) value of SRMR that was acceptable below the threshold. Moreover, to assess the value of *R*^2^, the structural model explained (17%) variance in employee engagement, (22%) variance in affective organizational commitment, and (26%) variance in job performance. As suggested by prior researchers, the value of *R*^2^ and Q^2^ should be > 0.10 or zero (Chin, 2010). Furthermore, Figure 2 results indicate that the values of *R*^2^ and Q^2^ are greater than the threshold value. Hence, the structural model was acceptable and met the criteria for further analysis.

**FIGURE 3:**
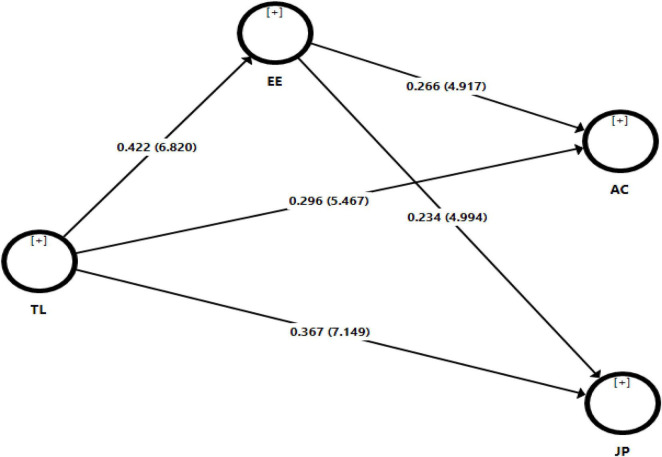
Structural model (*t*-values).

Furthermore, we tested hypotheses relationships and results are shown in Table 4 and Figure 3. To test H1, we found that transformational leadership had a positive and significant effect on affective organizational commitment (β = 0.296, *t* = 5.467, *p* = 0.001). Therefore, H1 was accepted. Meanwhile, we tested H2 and found that transformational leadership had a significant impact on job performance (β = 0.367, *t* = 7.149, *p* = 0.001). Consequently, H2 was supported. In addition, we hypothesized H3 and results indicate that transformational leadership had a positive influence on employee engagement (β = 0.422*, t* = 6.820, *p* = 0.001). Thus, H3 was accepted. Besides, we tested H4 and the results illustrate that employee engagement had a positive effect on affective organizational commitment (β = 0.266, *t* = 4.917, *p* = 0.001). So, H4 was supported. At last, H5 findings show that employee engagement had a significant impact on job performance (β = 0.234, *t* = 4.994, *p* = 0.001). Hence, H5 was also accepted.

**TABLE 4:** Table Path coefficients (direct effects).

Hypotheses	Relationships	β	T	*p*	*R*^2^ = EE 0.178; AC = 0.225; JP = 0.261 Q^2^ = EE 0.145; AC = 0.151; JP = 0.191
H1	TL—> AC	0.296	5.467	0.001	
H2	TL—> JP	0.367	7.149	0.001	
H3	TL—> EE	0.422	6.820	0.001	
H4	EE—> AC	0.266	4.917	0.001	
H5	EE—> JP	0.234	4.994	0.001	

*TL, Transformational leadership; EE, Employee engagement; AC, Affective organizational commitment; JP, Job performance.*

### Mediation Analysis

To test the indirect effect of employee engagement in the relationship between transformational leadership, affective organizational commitment, and job performance, the results of H6 and H7 are given in Table 5, which indicates that employee engagement had an indirect positive influence on the relationship between transformational leadership, affective organizational commitment, and job performance with standardized path coefficients (β = 0.112, *t* = 3.445, *p* = 0.001; β = 0.099, *t* = 3.399, *p* = 0.001). Likewise, to assess partial and full mediation effect, we followed the (Hair et al., 2012) approach using the variance accounted for (VAF) and analyzed the direct, indirect, and total effects. According to this method, if the value of VAF is between 20 and 80%, it presents partial mediation and if the value of VAF is more than 80%, there is full mediation that exists between the variables. Thus, Table 6 findings show that the value of VAF is below 80%, which presents partial mediation. Hence, H6 and H7 were accepted.

**TABLE 5:** Specific indirect effects.

Hypotheses	Relationship	β	t	*p*
H6	TL- > EE- > AC	0.112	3.445	0.001
H7	TL- > EE- > JP	0.099	3.399	0.000

*TL, Transformational leadership; EE, Employee engagement; AC, Affective organizational commitment; JP, Job performance.*

**TABLE 6:** Mediation analysis (employee engagement as a mediator).

Exogenous variable	Direct effect	Indirect effect	Total effect	VAF (%)	Mediation	Endogenous variable
TL	0.296	0.112	0.408	27.5%	Partial mediation	AC
TL	0.367	0.099	0.466	21.2%	Partial mediation	JP

*TL, Transformational leadership; EE, Employee engagement; AC, Affective organizational commitment, JP, Job performance.*

## Discussion

This study attempts to identify the role of transformational leadership as a key variable along with two dependent variables affective organizational commitment and job performance with the mediating effect of employee engagement. All the hypotheses results were verified. Prior studies revealed that transformational leadership and job performance positively affect employee engagement and affective organizational commitment (Schwarz, 2017; Yang et al., 2020). This result is also in line with prior researchers who argue that the transformational leaders help each other to advance a higher level of morale and motivation that engage employees in the organization (Sahu et al., 2018; Yang et al., 2020). This study finding demonstrated that employee engagement has an essential predictor and transformational leadership could improve employee affective organizational commitment and job performance if the intensity of employees’ engagement is higher in the organizations (Jena et al., 2018). The findings of prior study Lai et al. (2020) found a partial mediating effect of employee engagement in the relationship between transformational leadership and job performance. A previous study found transformative leadership had no direct effect on job performance (Ribeiro et al., 2018) it should be mentioned that to improve job performance more emphasis should be paid to supporting an engaged workforce. Results verify regarding employee engagement role is crucial for the transformational leadership behavior and performance outcome of employees, reliable with prior researcher’s findings (Le and Lei, 2017; Buil et al., 2019).

### Theoretical Implications

Transformational leadership is influenced by numerous theoretical frameworks, such as leadership and followership and SET that focus on two-way communication among leaders and followers to build mutual respect. In this study, we have confirmed that employee engagement, their level of affective commitment, and job performance are related to supportive leadership, mutually respectful relationships, and positive group processes in organizations. Whereas previous studies investigated the positive relationship of transformational leadership with employee attitude and performance separately (Ribeiro et al., 2018; Lai et al., 2020), this study confirmed that transformational leadership is the key catalyst in both encouraging employee affective commitment and job performance throughout the mediator, employee engagement. Moreover, based on the job demand resources (JD-R), results also specify that transformational leadership is an imperative job resource that influences employee work-related behavior (Katou et al., 2021). Furthermore, our results signify the action of a transformational leader is measured such as encouraging resources of job. Reciprocal positive relations among leaders and subordinates could bring constructive links in our structure of the model.

### Managerial Implications

This study provides practical contributions for policymakers, researchers, and hospitality managers. At first, it is concluded that leaders in an organization exhibit transformational leadership, which affects the perception of the employee to their responsibility and work resultantly direct to high-affective organizational commitment and job performance. So, hospitality managers require multilevel intervention leadership sessions or programs, one on one instruction for the establishment of required skills of transformational leadership for supporting workforce motivation, out-of-the-box thinking, and employee’s well-being. Being the holder of such kind of association, leaders improve skills of the transformational leadership that might expect to enhance motivation, engagement, and performance level of employees.

Second, various studies illustrated many factors that could develop employee engagement; this study attempt centered explicitly behavior and attitude of a leader that could support employees to be engaged in their work. To build transformational leadership associations, the organization will be able to establish significant results out as engaged employees. To develop better supervisory relations, staff connections help to support commitment toward the organization. For an instant, when employee and leader coach conferences create, it could bring trust and motivation among employees to achieve competitive advantage.

Third, this study results revealed the mediating role of employee engagement as crucial to support leader and employee outcome. So, it is an organizational requirement to establish a conducive atmosphere for work that upholds employee engagement. Specifically, practitioners of the hospitality industry could contribute to a constructive work environment to support employees, trust, and honor. Besides this, organizations can offer numerous resources of the job such as support from supervisor, the autonomy of the job, and positive feedback which could advance employee engagement. Hospitality industry managers could be helpful for the organizations to identify job resource value and discover for management to enhance job autonomy and meeting regarding employee successful performance.

### Limitations and Future Research Directions

This study argued that proper leadership could support and guide employees to engage in work; however, we do not know what kinds of other leadership behaviors are appropriate to facilitate employee engagement, commitment, and performance. Future researchers can examine other leadership styles (e.g., empowering leadership) that may impact employee engagement by adding those into the structural model. In addition, this study was conducted with organizations in China. The effects of differences in practice, organizational culture, and national culture may impact the results accordingly. Moreover, future research could explore other factors as turnover intention, conflict, and stress to investigate their effect on the relationship between transformational leadership and employee engagement.

Furthermore, this study result is based on the cross-sectional data and data were gathered from the two provinces of China, future research could use a method of longitudinal design to conduct a study among transformational leadership and insight of subordinate attitude, the performance of job, and engagement with large sample size. The relationship between affective organizational commitment and job performance was not tested in this study. A future study could test this relationship with the different samples. In addition, this study examined only the affective organizational commitment element and did not cover the other two factors of organizational commitment, e.g., continuance and normative commitment. Each factor could have a dissimilar influence on employee job-related behavior and performance. The addition of the other two components might have different results. According to Dai et al. (2013), future researchers could examine a comparison of ethical, transactional, and transformational leadership regarding job performance and employee engagement.

## Data Availability Statement

The raw data supporting the conclusions of this article will be made available by the authors, without undue reservation.

## Ethics Statement

The studies involving human participants were reviewed and approved by the Jiangsu University, China. The patients/participants provided their written informed consent to participate in this study.

## Author Contributions

MM and MA proposed the research, analyzed the experimental results, and wrote the manuscript. ZW and FG designed and carried out the experiments. WJ and SG extensively edited and revised the manuscript. All authors contributed to the article and approved the submitted version of the manuscript.

## Conflict of Interest

The authors declare that the research was conducted in the absence of any commercial or financial relationships that could be construed as a potential conflict of interest.

## Publisher’s Note

All claims expressed in this article are solely those of the authors and do not necessarily represent those of their affiliated organizations, or those of the publisher, the editors and the reviewers. Any product that may be evaluated in this article, or claim that may be made by its manufacturer, is not guaranteed or endorsed by the publisher.
